# Sublingual Delivery of Frovatriptan: An Indication of Potential Alternative Route

**DOI:** 10.1155/2014/675868

**Published:** 2014-10-29

**Authors:** Hitesh Verma, Surajpal Verma, Shyam Baboo Prasad, Harmanpreet Singh

**Affiliations:** School of Pharmaceutical Sciences, Lovely Faculty of Applied Medical Sciences, Lovely Professional University, Phagwara, Punjab 144411, India

## Abstract

Frovatriptan, a 5-HT_1B_ and 5-HT_1D_ receptor agonist, is used for the treatment of acute migraine attack. This molecule is classified into second line therapy because of its slow onset of action (peak response obtained after 4 hours of administration) and low bioavailability (25%). Moreover, its therapy is the most costly among all triptans. Attempt has been made in present work to suggest a way out to fasten its onset of action and to enhance its bioavailability. Prepared tablets were evaluated by physicochemical tests, *in vitro* permeation studies, *ex vivo* permeation studies, and histopathological studies. Suitable mathematical calculations were performed to calculate the minimum amount of bioavailability that could be enhanced. Tablets containing chitosan (5% w/w) were found to give optimum results. Prepared tablets can double the bioavailability of frovatriptan and can initiate its response within 10 minutes of its administration. Suggestive alternative has the potential to increase the efficacy of frovatriptan for treating acute migraine attack.

## 1. Introduction

According to International Headache Society (IHS), acute migraine attack is a deliberating cerebrovascular disorder characterized by throbbing unilateral (though, in some cases, can be bilateral), pulsatile, and moderate to severe intensity pain which is often associated with incidences of nausea, vomiting, photophobia, and phonophobia. This pain is aggravated by physical activity of the patient. An untreated migraine attack can persist for 4 to 72 hours and significantly affect the quality of life of migraineur [1–5]. In addition, migraine exhibits sexual dimorphism with a prevalence ratio of 7 : 3 among females and males, after the attainment of puberty [6]. As per IHS, an ideal migraine therapy should focus on regaining the functional ability of patient as soon as possible in a cost-effective manner and therapeutic effect should last for longer duration. Such therapy will alleviate the patient fear to get migraine attack back and will help in improving their quality of life [1, 2].

Triptans, 5-HT_1B_ and 5-HT_1D_ receptor agonists, are considered to be the first line therapy for treating the migraine attack. This therapeutic class comprises seven members, namely, sumatriptan, zolmitriptan, rizatriptan, eletriptan, almotriptan, naratriptan, and the latest one “frovatriptan.” All triptans share similar pharmacodynamics but each of them comprises a different pharmacokinetic profile which makes each of them a unique of its kind. According to current clinical practice and IHS, triptans can be broadly classified into two main categories based on efficacy to achieve pain-free response after 2 hours of* per oral* administration of dosage form, namely, high efficacy triptans comprising sumatriptan, zolmitriptan, rizatriptan, eletriptan, and almotriptan and low efficacy triptans comprising naratriptan and frovatriptan [7–9].

Though frovatriptan has the highest activity for 5-HT_1B_ receptor (8.2 out of 10) and third highest activity for 5-HT_1D_ receptors with the most favourable reoccurrence and adverse drug reaction profile among all the triptans, it is still classified as low efficacy triptan [1]. This is due to its pharmacokinetic limitations, namely, (i) slow onset of action (peak response achieved at 4th hour after the administration of the dosage form) and (ii) low bioavailability (due to its metabolism by hepatic enzyme CYP1A2). These limitations result in underutilization of this highly potent molecule, even though its elimination half-life is the highest among all triptans (26 hours). It is available in market as film-coated tablet (containing 2.5 mg frovatriptan) intended to be administered by* per oral *route [1, 7].* Per oral* administration is not generally acceptable by migraineur as attack is often associated with nausea and vomiting. Therefore, an alternative route of administration is always a focus of migraine research [2, 9]. Moreover,* per oral* therapy of frovatriptan is reported to be the most costly therapy among all triptans (in terms of number of doses required to take for achieving pain-free response) with a cost-effective ratio of $162.49 [10]. Attempt had been made for delivery of frovatriptan by nasal route [11] but this delivery system is often associated with sensitization problems and requires highly sterile conditions to be maintained at industrial scale. Nothing can replace the patient's acceptability towards oral drug delivery because it does not require assistance in usage and free from sterilization problems [12].

Clinically, sublingual delivery is reported to be the most promising alternative route for enhancing the bioavailability and fastening the onset of action in comparison to its oral equivalents because it has high blood supply, a very thin membranous barrier (190 *μ*m), and an ability to bypass hepatic first pass metabolism [13]. Therefore, it could be a promising route for the administration of frovatriptan for treating acute migraine attack. But sublingual administration of any drug has one major hurdle; that is, sublingual mucosa is continuously flushed out by saliva at the rate of 0.3 mL/minute under resting condition and 1.0 mL/minute under stimulated conditions [14]. The dosage form tends to be involuntary swallowed, completely or partially, thereby decreasing its residence time at the site of absorption. In order to avoid this problem, it is utmost important to have some adhesive component in formulation. Bioadhesive polymers commonly used for this purpose are carbomer, cellulose derived polymers, and chitin derived polymers [13, 15, 16].

In the present study, an attempt has been made to develop sublingual fast disintegrating tablets of frovatriptan using different bioadhesive polymers. The concentration of bioadhesive polymers was chosen without significantly affecting the basic tablets characteristics (i.e.,* in vitro* dispersion and dissolution time). To ensure this, tablets were subjected to basic physical tests of tablets. In addition, frovatriptan sublingual tablets were also evaluated for* in vitro* permeation through cellophane membrane,* ex vivo* permeation through ventral surface of tongue as well as through floor of mouth of porcine mucosa, and histopathological characterization. Finally, mathematical correlation has been established to calculate minimum amount of bioavailability that is supposed to be enhanced through sublingual administration of frovatriptan.

## 2. Materials and Methods

### 2.1. Materials

Frovatriptan (FSM) was obtained as a gift sample from Azakem Chemicals Pvt. Ltd. (Hyderabad, India). Chitosan (medium viscosity grade) was obtained as gift sample from Shanghai Biochemicals Pvt. Ltd. (Shanghai, China). Microcrystalline cellulose, that is, MCC 102 (Avicel 102), was obtained as gift sample from Arihant Trading Co. (Mumbai, India). Sodium starch glycolate (SSG) and Spray-dried lactose (SDL) were obtained as gift samples from Signet Chemical Corporation Pvt. Ltd. (Mumbai, India). Sodium carboxymethyl cellulose, that is, NaCMC (viscosity of 4% w/v aqueous solution at 25°C: 50–200 cps), and hydroxypropyl methyl cellulose, that is, HPMC K4M (viscosity of 2% w/v aqueous solution at 25°C: 4000 cps), were procured from Central Drug House (New Delhi, India). Colloidal silicon dioxide (CSD) and magnesium stearate (MS) were purchased from SD Fine Chemicals Pvt. Ltd. (Mumbai, India).

### 2.2. Formulation of Frovatriptan Sublingual Tablets

#### 2.2.1. Optimization of Concentration of Superdisintegrant

According to literature, SSG can be used as disintegrant in tablet dosage form in range of 2–8% w/w. According to the same reference, best results will be obtained with 4% w/w but in many cases 2% w/w may be enough [17]. Every formulation is unique of its kind; therefore, based on literature review, we cannot select a specific concentration of superdisintegrant. Hence, optimization was done over entire range of permissible uses. Tablets were prepared as per formulae mentioned in Table 1.

Materials were individually weighed, sieved, and mixed by adopting geometrical dilution technique. Final blend was directly compressed into tablets at a constant compression force using multistation tablet compression machine (Trover Pharmamach, India) fitted with biconvex dies and punches having diameter of 6 mm. Optimum concentration of superdisintegrant was selected based on* in vitro* dispersion test developed and validated by us. In a test tube, 2 mL of phosphate buffer (pH 6.8) equilibrated at 37 ± 0.5°C was taken and a tablet was dropped into it. Test tube was rotated gently in mechanical shaker to slightly stir the liquid. Time taken by tablet to disperse completely was recorded as* in vitro* dispersion time.

#### 2.2.2. Optimization of Type and Concentration of Bioadhesive Polymer

Based on results obtained in Section 2.2.1, SSG 2% w/w was found to give optimum results. Therefore, by considering 2% w/w concentration of SSG, further optimization was done for selection of type and concentration of bioadhesive polymer. Different batches were manufactured as per formulae mentioned in Table 2. Tablets were prepared by adopting the same procedure as mentioned in Section 2.2.1. One control batch was also prepared containing only superdisintegrant but no bioadhesive polymer for comparison purpose (equivalent to C2 formula of Table 1). According to literature, 0.5–5.0% of bioadhesive polymers can provide sufficient bioadhesion to the particles generated from fast disintegrating dosage form without affecting the results of physicochemical parameters, particularly,* in vitro *dispersion time and* in vitro* dissolution time to statistically significant extent [15]. Therefore, this concentration range was used in optimization procedure.

Prepared tablets were evaluated for different physiochemical parameters, namely, content uniformity, weight variation, friability, hardness, thickness,* in vitro* dispersion time, and* in vitro* dissolution time.

Content uniformity was analysed by withdrawing twenty tablets randomly from each batch and crushing them in a mortar pestle. A powder equivalent to average weight of twenty tablets was withdrawn and dissolved in distilled water followed by filtration through 0.22 *μ*m nylon filter. Filtrate was subjected to necessary dilutions and was analysed by using UV-visible spectrophotometer (Shimadzu Co. Ltd., Japan) at 244 nm [18].

Weight variation was performed by withdrawing twenty tablets randomly from each batch and percent weight variation was calculated and analysed using USP criteria [19].

Friability was checked for determining the tablet resistance towards chipping and abrasion while handling. It was tested by using Roche type friabilator (Popular Traders, India) rotating at a speed of 25 revolutions per minute. Preweighed tablet sample from each batch was subjected to 100 revolutions and was reweighed after dedusting. Percent friability was calculated for each batch.

Hardness of tablets was determined by using Monsanto hardness tester (Popular Traders, India). Randomly ten tablets were withdrawn from each batch and hardness of each tablet was determined as results were reported in terms of mean ± standard deviation (SD).

Thickness of tablets was determined from each batch by using digital micrometer (Mitutoyo, Japan) by placing the tablet in between spindle and anvil in perpendicular direction to the micrometer. Thickness was determined in replicate of ten and results were reported as mean ± SD.

*In vitro* dispersion time was determined by using the same procedure mentioned in Section 2.2.1. Results were determined in replicate of six and were reported as mean ± SD.

Tablets from each batch were evaluated for drug dissolution using USP II (paddle type) dissolution apparatus (Labindia, India) and using phosphate buffer pH 6.8 at 37 ± 0.5°C. Samples were withdrawn at specified time intervals (2, 4, 8, 16, and 32 minutes). Withdrawn samples were filtered through 0.22 *μ*m nylon filter and analysed by using UV-visible spectrophotometer at 244 nm [18]. Dissolution profiles of different batches were compared using student unpaired *t*-test (*P* < 0.05).

*In vitro* permeation studies were performed by using Franz diffusion cell (Singh Scientific Pvt. Ltd., India) and using cellophane membrane as a permeability barrier. In donor compartment, 2 mL of phosphate buffer (pH 6.8) at 37 ± 0.5°C was taken as dissolution media which simulate the physiological conditions in terms of pH and volume. In receptor compartment, phosphate buffer (pH 7.4) at 37 ± 0.5°C was taken as media which simulate the plasma pH. A tablet from each batch was placed in donor compartment, which disintegrates in available media to release frovatriptan. Released drug was passed through the membrane barrier and entered the receptor compartment. Samples were withdrawn at specified time intervals (2, 5, 10, 20, 30, 45, 60, 90, 105, and 120 minutes) and analysed by UV-visible spectrophotometer at 244 nm [18].

A plot was drawn between cumulative amount of drug released per unit area (*y*-axis) and time (*x*-axis) for each batch and from the linear portion of the plot, steady state flux (*J*_ss_) was calculated. From *J*_ss_, permeability coefficient (*K*_*p*_) was calculated by using the following expression:

(1) $$\begin{array}{c} K_{p}=\frac{J_{\text{ss}}}{C_{v}}, \end{array}$$

where *C*_*v*_ is the concentration of frovatriptan in donor compartment [15, 16].

Based on results obtained from* in vitro* dissolution studies and* in vitro* permeation studies through cellophane membrane, batches showing promising results were evaluated for* ex vivo *permeability studies through porcine sublingual mucosa (mucosa of ventral surface of tongue and mucosa of floor of mouth). Freshly excised oral cavity was brought in laboratory premises through local slaughter house in 10% formalin solution and mucosa was removed using scalper blade. Experimental conditions for* ex vivo* permeation studies were the same as those adopted for* in vitro* permeation studies. There were two experimental setups for every batch. In one set, permeability of released drug was studied from ventral surface of tongue, while in another set permeability was studied across mucosa of floor of mouth. Samples were withdrawn at specified time intervals (2, 5, 10, 20, 30, 45, 60, 90, 105, and 120 minutes) and were analysed by UV-visible spectroscopy at 244 nm [18]. Similar calculations were performed for calculating *K*_*p*_ as described for* in vitro* permeation studies.

Permeability ratios were determined among ventral surface of tongue and floor of mouth to establish a correlation because after taking a tablet through sublingual route it will be simultaneously exposed to both types of mucosa; total drug absorption through sublingual mucosa was calculated as sum of drug permeated through mucosa of ventral surface of tongue and mucosa of floor of mouth.

Histopathological studies were performed for optimised formulation by exposing it to both types of mucosa (mucosa of ventral surface of tongue and mucosa of floor of mouth) moistened with phosphate buffer (pH 6.8) for 2 hours. Whole experimental assembly was maintained at 37 ± 0.5°C. A control assembly was also established in which both mucosae were exposed to only phosphate buffer (pH 6.8). At the end of 2 hours, mucosae were removed from both test and control assemblies thoroughly washed with phosphate buffer (pH 6.8) and were stored in 10% formalin solution till further histopathological evaluation. Mucosae were dehydrated with ethanol and were fixed in paraffin wax. Sectioning of embedded tissue was done followed by staining with hematoxylin and eosin and was observed under binocular microscope (Nikon, Japan).

## 3. Results and Discussion

### 3.1. Optimization of Concentration of Superdisintegrant

**Figure 1:**
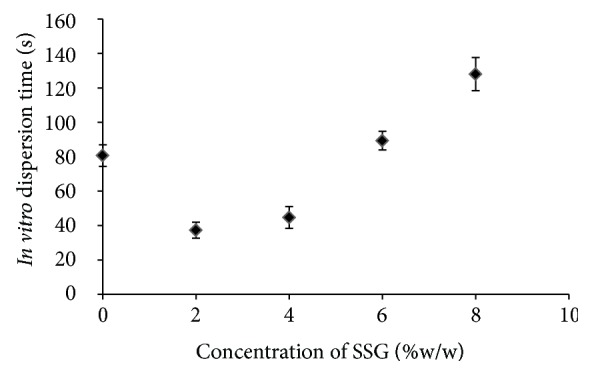
Effect of concentration of SSG (% w/w) on* in vitro* dispersion time.

SSG was selected as superdisintegrant based on the availability of material at the time of experimentation. During optimisation procedure, it was observed that, as we increase the concentration of SSG, the* in vitro *dispersion time increases to a statistically significant extent (*P* < 0.05; student unpaired *t*-test). SSG in high concentration (particularly at 6% and 8% w/w concentration) forms gelatinous barrier around the tablet core which hinders in further imbibition of dispersion media (Figure 1). Similar type of results was also obtained for aceclofenac fast disintegrating tablets by Setty et al. [20]. Results of batch containing 2% w/w and 4% w/w SSG did not vary to statistically significant extent (*P* < 0.05; student unpaired *t*-test). Superdisintegrant is a swellable ingredient not a soluble one [21] and its presence in excess can result in grittiness; therefore, we decided to incorporate its least required amount in the formulation (2% w/w).

### 3.2. Optimization of Type and Concentration of Bioadhesive Polymers

Content uniformity of batches containing different bioadhesive polymers at variable concentration ranges from 98.06% to 101.53% depicting that there is uniform distribution of frovatriptan throughout the blend (Table 3).

Weight variation analysis was performed as per USP [19] and was found to lie within the acceptable limit of ±10% of the average weight obtained in each case, which ensures the uniformity of die fill during the manufacturing of batch. It ensures that powder is having good flow characteristics. Data associated with weight variation analysis is reported in Table 3.

Thickness was found to lie in acceptable limit of ±5% in all the batches [22]. Associated data with the same is mentioned in Table 3.

Hardness and friability are two such parameters which describe the mechanical strength of tablet and its resistance towards chipping and abrasion during the course of handling. Hardness and friability of batches range from 5.01 ± 2.01 to 6.62 ± 0.34 Kg cm^−2^ and 0.00 to 0.70%, respectively. According to literature, friability of orodispersible tablet should not be >1.0% [23]. Hardness is not a compendial parameter and there are no well-defined limits of it available in literature for orodispersible tablets. According to [24], an immediate release tablet is said to have good mechanical strength if it has hardness >4 Kg cm^−2^. Therefore, we prepared tablets having hardness more than this limit provided that it should not affect* in vitro* dispersion time and* in vitro* dissolution time. Our results did not represent any effect of hardness on the same. It might be because of presence of hydrophilic excipients in the formulation.

**Figure 2:**
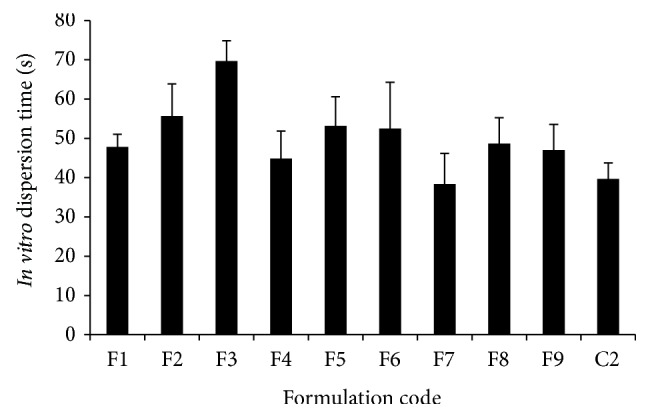
*In vitro* dispersion time of batches containing different bioadhesive polymers in variable concentration.

Since compendial disintegration apparatus did not simulate the physiological sublingual conditions, we developed and used* in vitro* dispersion technique mentioned in Section 2.2.1. Presence of bioadhesive polymers did not significantly affect the* in vitro* dispersion time in comparison to control batch. No correlation was observed with respect to concentration of chitosan and HPMC K4M with* in vitro* dispersion time. In case of NaCMC, though* in vitro* dispersion time increased with the increase in its concentration, the difference was found to be statistically insignificant (*P* > 0.05).  Figure 2 depicts results of* in vitro* dispersion time of various batches. For control, NaCMC, HPMC K4M, and chitosan containing batches,* in vitro* dispersion time was found to be 39.67 ± 4.08, 47.83 ± 3.25 to 69.67 ± 5.16, 44.83 ± 7.00 to 53.17 ± 7.44, and 38.33 ± 7.84 to 48.67 ± 6.59, respectively.

From results of* in vitro* dissolution studies (Table 4), it was evident that, within 4 minutes of starting the experiment, more than 90% of the drug was dissolved in surrounding media, except for the formulations containing 5% w/w level of NaCMC (F3). Difference was found to be statistically significant (*P* < 0.05). Results comply with the* in vitro* dissolution parameters of sublingual tablets; that is, they should release more than 80% of the drug content within 15 minutes of initiation of experiment [14].

Two major factors which influence the* in vitro* dissolution time of tablet are method adopted for manufacturing of tablet and presence of hydrophilic excipients within the tablet. Tablets produced by direct compression technology are reported to yield fine powder after disintegration which have larger surface area and thus have faster dissolution rate. In addition, presence of lactose aids in dissolution due to its high water solubility [15, 21]. HPMC K4M is known to form release rate limiting gelatinous layer around the tablet core. But this phenomenon is observed when its concentration within the tablet is >10% w/w [25, 26]. Unmodified chitosan though has bioadhesive characteristic but its inability to control the release of drug is reported in literature [27]. But there was statistically significant difference in release rate of frovatriptan from tablets containing 5% w/w NaCMC, because at this concentration level, NaCMC forms gelatinous barrier around the tablet which increases the diffusional path of the drug, thereby decreasing its release rate [15].

**Figure 3:**
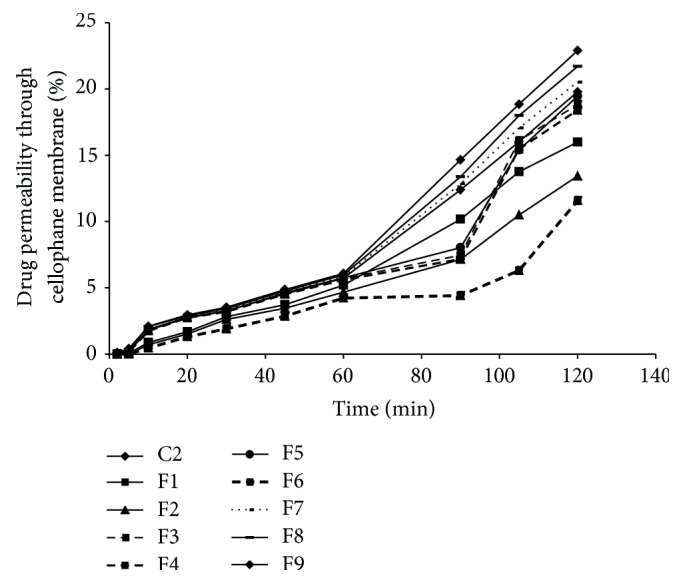
Permeation plot of sublingual tablets of frovatriptan containing SSG 2% w/w and bioadhesive polymers in variable concentrations through cellophane membrane (*n* = 3).

*In vitro* permeation of frovatriptan at the end of the second hour was found to range between 11.57 ± 0.29 and 22.89 ± 0.75% (Figure 3). Values of steady state flux and permeation rate constants are shown in Table 5. Permeation rate of frovatriptan was found to be slow in all the batches containing bioadhesive polymers because of limited volume of surrounding media in the donor compartment. In this limited volume, bioadhesive polymer present in the formulation hydrates and forms a gelatinous layer around the tablet core which increases the diffusional path of the drug. At this stage, diffusion becomes a rate limiting step [27]. NaCMC is reported to have high swelling index [28]. Because of this, tablets containing 5% w/w concentration of NaCMC show slower permeation rate (*P* < 0.05). Therefore, based on results of* in vitro* dissolution studies and* in vitro* permeation studies, F3 batch was removed from further optimization procedure.

**Figure 4:**
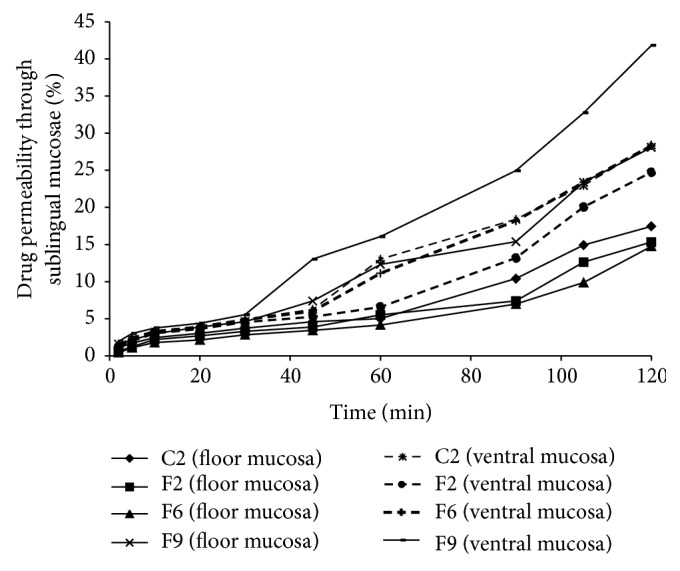
Permeation plot of sublingual tablets of frovatriptan across sublingual mucosae (*n* = 3).

If* in vitro* dispersion time and percent dissolution are not affected to statistically significant extent, it is wise to choose the maximum amount of bioadhesive polymer for increasing the residence time of dosage form at the site of absorption. Therefore, tablets containing 5% w/w level of chitosan and HPMC K4M and 2% w/w level of NaCMC were taken for carrying out* ex vivo* permeation studies through porcine sublingual pouch (Figure 4). Porcine was selected as animal model because its oral mucosa has biochemical and histological similarities with human oral mucosa [29, 30]. Whenever a sublingual tablet is taken by a patient, it is simultaneously exposed to both mucosa of ventral surface of tongue and mucosa of floor of mouth. Moreover, it was found that the permeability of both of the mucosae is not the same. Floor of mouth exhibits approximately half of the permeation rate of ventral surface of tongue [31]. Therefore, permeation studies should be carried out from both types of mucosa in order to establish valid correlation. The ratio of permeability coefficient of frovatriptan through mucosa of ventral surface of tongue to mucosa floor of mouth depicts 2 : 1 relationship (Table 6). It means that mucosa of ventral surface of tongue is two times more permeable than mucosa of floor of mouth. It can be attributed to its rich vasculature and very thin membranous barrier. Values of permeation coefficients revealed that chitosan acts as a permeation enhancer and batches containing 5% w/w concentration of chitosan (F9) were found to have higher* ex vivo* permeation rate as compared to other batches (*P* < 0.05). Permeation enhancer effect of chitosan was observed in case of both mucosa of ventral surface of tongue and mucosa of floor of mouth (Table 6). These results are supported by literature, as chitosan is reported to dissolve the tight junctions between the cells, thereby opening the paracellular route of drug transportation which is the major route of transportation of hydrophilic drugs [13].

**Figure 5:**
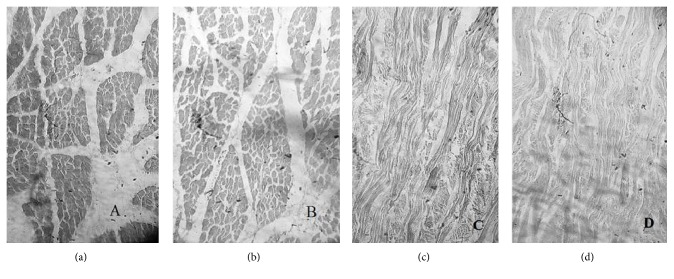
Histopathological characterization of test formulation (F9) with respect to control batch (a) mucosa of ventral surface of tongue exposed to test formulation, (b) mucosa of ventral surface of tongue—control, (c) mucosa of floor of mouth exposed to test formulation, and (d) mucosa of floor of mouth—control.

According to histopathological studies of F9 formulation, there is no difference between the gross histological characters of control samples and test samples (Figure 5). Therefore, the excipients of the formulation are biocompatible.

### 3.3. Theoretical Analysis of Permeation Data

Theoretical calculations were performed to determine minimum amount of bioavailability that could be enhanced by delivering frovatriptan through sublingual route. Such calculations were performed because of the limitation of resources available at the time of experimentation.* In vitro* bioadhesion time for F9 formulation was determined using modified disintegration test apparatus and was found to be 10.5 ± 3.2 minutes and 10.0 ± 1.8 minutes for mucosa of ventral surface of tongue and mucosa of floor of mouth, respectively. Since these values did not vary to statistically significant extent (student unpaired *t*-test; *P* < 0.05), a bioadhesion time of 10 minutes was taken as reference time point for calculation of minimum amount of enhanced bioavailability through sublingual route. After this time, it was assumed that the remaining drug will be involuntarily swallowed by the patient, which will tend to give a bioavailability of 25% (bioavailability of per oral dosage form). Calculation of theoretical amount of bioavailability can be explained as follows by taking the example of F9 batch.

Total amount of drug permeated within 10 minutes from F9 formulation across both types of sublingual mucosae was found to be 6.77 ± 2.60% (average consideration 6.77%, equivalent to 0.16925 mg of frovatriptan base). Now the overall surface area of both types of mucosa is 26.5 ± 4.2 cm^2^ [32]. Therefore, the observed permeation was extrapolated to 26.5 ± 4.2 cm^2^ area (26.5 cm^2^ was taken as reference value for calculation) which will give us an idea of total amount of drug that can permeate within 10 minutes through sublingual mucosae under physiological conditions. For F9, it was found to be 0.881 mg of the administered dose of free base. Rest of the amount of drug (1.619 mg) was presumed to be involuntary swallowed by the patient and will result in bioavailability of 25% (0.405 mg). Therefore, total bioavailability that could result by administering F9 formulation was calculated as 1.286 mg (51.44%).

Therefore, though during experimentation we lack resources but our permeation results depicted that sublingual administration of frovatriptan can result in double of its bioavailability, which can be proven clinically and is the matter of future research.

Moreover, as discussed in Section 1 of the present paper, delay in onset of action of frovatriptan is because of its pathophysiological limitation, that is, decreased gastrointestinal transit time during attack. Once frovatriptan enters the blood, it initiates its action because of its high affinity towards 5-HT_1B_ and 5-HT_1D_ receptors. As only 0.625 mg can initiate the response (though after 4 hours of administration) in case of per oral therapy, it means that only the amount of drug permeated through sublingual mucosa (0.881 mg) can onset the action. Hence, we can expect the action of frovatriptan within 10 minutes of its administration through sublingual route.

Therefore, considering the points mentioned above, our research could lay down a base for future research that could enhance the efficacy of frovatriptan. Such delivery could enhance the bioavailability as well as fasten its onset of action. In turn, it will decrease the number of doses required to be taken to alleviate the pain and will decrease the overall cost of the therapy.

## 4. Conclusion

Frovatriptan though has the potential to alleviate the suffering of migraineur but its current dosage form available in market is not efficient which discourages the use of this molecule as first line therapy for treatment of acute migraine attack. Present research was aimed at suggesting sublingual route as potential alternative route of its administration which can enhance its efficacy. Prepared tablets were found to have good physicochemical properties and short* in vitro* dispersion and dissolution time.* Ex vivo* permeation studies reveal that F9 formulation (containing 5% w/w chitosan) has the potential to double the bioavailability and can result in onset of action within 10 minutes of administration. It might be attributed to permeation enhancer effect of chitosan. Though present research did not incorporate* in vivo* studies to validate the results due to unavailability of resources at the time of experimentation but it certainly pointed out the importance of sublingual delivery of frovatriptan which can lay down the foundation for future research. Prepared formulations were also found to be biocompatible as revealed by histopathological studies.

## Figures and Tables

**Table 1:** Optimization of concentration of superdisintegrant.

Ingredients	Amount (mg/tablet)				
	C1	C2	C3	C4	C5
FSM	3.91	3.91	3.91	3.91	3.91
SSG	0.00	1.70	3.40	5.10	6.80
SDL	54.98	53.79	52.60	51.41	50.22
MCC 102	23.56	23.05	22.54	22.03	21.52
CSD	1.70	1.70	1.70	1.70	1.70
MS	0.85	0.85	0.85	0.85	0.85

Total weight (mg)	85	85	85	85	85

**Table 2:** Optimization of type and concentration of bioadhesive polymer.

Ingredients	Amount (mg/tablet)									
	F1	F2	F3	F4	F5	F6	F7	F8	F9	C2
FSM	3.91	3.91	3.91	3.91	3.91	3.91	3.91	3.91	3.91	3.91
SSG	1.70	1.70	1.70	1.70	1.70	1.70	1.70	1.70	1.70	1.70
NaCMC	0.43	1.70	4.25	—	—	—	—	—	—	—
HPMC K4M	—	—	—	0.43	1.70	4.25	—	—	—	—
Chitosan	—	—	—	—	—	—	0.43	1.70	4.25	—
SDL	53.49	52.60	50.81	53.49	52.60	50.81	53.49	52.60	50.81	53.788
MCC 102	22.92	22.54	21.78	22.92	22.54	21.78	22.92	22.54	21.78	23.052
CSD	1.70	1.70	1.70	1.70	1.70	1.70	1.70	1.70	1.70	1.70
MS	0.85	0.85	0.85	0.85	0.85	0.85	0.85	0.85	0.85	0.85

Total weight (mg)	85.00	85.00	85.00	85.00	85.00	85.00	85.00	85.00	85.00	85.00

**Table 3:** Physicochemical parameters of batches containing SSG (2% w/w) and various bioadhesive polymers.

Batch code	Content uniformity (%)	Weight variation (mg) (mean ± SD)	Hardness (Kg cm^−2^) (*n* = 10) (mean ± SD)	Thickness (mm) (*n* = 10) (mean ± SD)	Friability (%)
F1	99.88	87.90 ± 3.01	5.21 ± 0.02	3.73 ± 0.05	0.12
F2	98.52	85.60 ± 3.98	6.11 ± 0.23	3.69 ± 0.06	0.70
F3	98.59	87.00 ± 3.96	5.60 ± 0.39	3.66 ± 0.05	0.00
F4	99.92	87.00 ± 4.17	5.55 ± 1.78	3.72 ± 0.04	0.69
F5	101.18	86.80 ± 3.98	5.81 ± 0.09	3.66 ± 0.05	0.00
F6	100.87	86.85 ± 3.51	6.62 ± 0.34	3.71 ± 0.06	0.23
F7	98.06	86.25 ± 4.42	5.01 ± 2.01	3.69 ± 0.06	0.12
F8	98.41	86.30 ± 3.73	5.79 ± 0.11	3.71 ± 0.03	0.33
F9	101.53	86.25 ± 3.85	5.50 ± 0.72	3.68 ± 0.04	0.00
C2	99.368	86.10 ± 4.29	6.19 ± 0.88	3.67 ± 0.05	0.12

**Table 4:** Dissolution values (% ±S.D.) of sublingual tablets of frovatriptan containing SSG 2% w/w and bioadhesive polymers in variable concentrations (*n* = 6).

Batch code	Time (minutes)					
	0	2	4	8	16	32
C2	0.00	94.08 ± 0.05	96.36 ± 0.08	97.12 ± 0.05	98.34 ± 0.07	98.95 ± 0.04
F1	0.00	90.86 ± 0.01	94.66 ± 0.02	99.45 ± 0.01	102.30 ± 0.01	104.01 ± 0.01
F2	0.00	79.13 ± 0.06	94.34 ± 0.08	104.32 ± 0.03	105.39 ± 0.06	105.98 ± 0.05
F3	0.00	57.43 ± 0.06	64.84 ± 0.03	85.84 ± 0.09	105.31 ± 0.05	106.23 ± 0.07
F4	0.00	80.78 ± 0.19	96.35 ± 0.14	105.26 ± 0.09	105.92 ± 0.05	106.03 ± 0.05
F5	0.00	76.76 ± 0.05	95.91 ± 0.01	105.46 ± 0.26	104.82 ± 0.31	106.31 ± 0.07
F6	0.00	76.45 ± 0.01	93.62 ± 0.26	101.56 ± 0.12	105.26 ± 0.34	106.05 ± 0.87
F7	0.00	87.73 ± 0.01	103.91 ± 0.06	105.44 ± 0.13	106.10 ± 0.17	106.27 ± 0.33
F8	0.00	79.42 ± 0.16	96.97 ± 0.03	105.14 ± 0.02	106.32 ± 0.05	107.06 ± 0.14
F9	0.00	80.67 ± 0.07	92.05 ± 0.06	100.49 ± 0.01	105.81 ± 0.15	106.42 ± 0.02

**Table 5:** Steady state flux (*J*_ss_) and permeability coefficient (*K*_*p*_) values of frovatriptan through cellophane membrane at the end of 2 hours.

Batch code	C2	F1	F2	F3	F4	F5	F6	F7	F8	F9
*J*_SS_ (mg cm^−2^min^−1^)	0.157	0.133	0.105	0.077	0.142	0.143	0.137	0.165	0.173	0.184
*K*_*p*_ (cm min^−1^)	0.126	0.106	0.084	0.062	0.113	0.114	0.110	0.132	0.138	0.147

**Table 6:** Steady state flux (*J*_ss_) and permeability coefficient (*K*_*p*_) values of frovatriptan through sublingual mucosae at the end of 2 hours [V = mucosa of ventral surface of tongue; F = mucosa of floor of moth; ratio = *K*_*p*_ of V/*K*_*p*_ of F].

Batch code (→)		C2	F2	F6	F9
*J*_ss_ (mg cm^−2^min^−1^)	V	0.22	0.18	0.22	0.32
	F	0.13	0.11	0.10	0.21
*K*_*p*_ (cm min^−1^)	V	0.18	0.14	0.18	0.26
	F	0.10	0.09	0.08	0.17
Ratio (→)		1.80	1.56	2.25	1.53
